# Factors Influencing Physicians’ Perceived Compensation Satisfaction in China: Cross-Sectional Study

**DOI:** 10.2196/85936

**Published:** 2026-02-10

**Authors:** Wei Wei, Ning Hu, Jing Ma, Xiaoying Jiang, Aiping Zhu, Chunyu Zhang

**Affiliations:** 1 Youth League Committee Linyi People's Hospital Linyi China; 2 School of Population and Health Renmin University of China Beijing China; 3 Emergency Department Ningxia Hui Autonomous Region Peoples Hospital Yinchuan China; 4 Department of Science Beijing Chest Hospital Beijing China; 5 Hospital Office China-Japan Friendship Hospital Beijing China; 6 National Office for Chronic Respiratory Disease Prevention and Control China-Japan Friendship Hospital Beijing China

**Keywords:** compensation perceptions, compensation satisfaction, qualitative comparative analysis, compensation scheme transparency, junior physician, senior physicians, Chinese physicians, health care quality

## Abstract

**Background:**

Physicians’ perceived compensation satisfaction enhances work enthusiasm, ensures health care system stability, and inspires health care quality. However, few studies have investigated the combined effect of multiple influencing factors on this perception.

**Objective:**

This study investigates the factors influencing Chinese physicians’ perceived compensation satisfaction.

**Methods:**

A cross-sectional survey was conducted between April and May 2024 to examine physicians’ perceived compensation satisfaction, alongside their sociodemographic characteristics and perceived transparency of compensation schemes. A total of 325 valid responses were obtained, with 163 male and 162 female participants. Qualitative comparative analysis was then employed to identify the factors associated with physicians’ perceived compensation satisfaction.

**Results:**

The analysis yielded two models: junior physicians’ perceptions and senior physicians’ perceptions. For junior physicians, compensation scheme transparency contributed to higher perceived compensation satisfaction, regardless of salary or work hours. For senior physicians, two paths contributed to perceived compensation satisfaction: (1) higher salary and compensation scheme transparency and (2) lower salary and compensation scheme transparency combined with a higher technical rank and fewer work hours.

**Conclusions:**

The determinants of perceived compensation satisfaction showed heterogeneity between junior and senior physicians, which underscores the necessity of formulating tiered, targeted compensation packages to improve their perceived compensation satisfaction.

## Introduction

Perceived compensation satisfaction constitutes a critical component of health care human resources management. Within the health care sector, physician compensation satisfaction is instrumental in safeguarding the stability of the health care system and enhancing interprofessional care coordination [1-3]. Notably, physicians’ satisfaction with their compensation exerts a significant impact on their work motivation: higher levels of compensation satisfaction facilitate the retention of elite medical talent and incentivize the delivery of high-quality clinical services. Conversely, low compensation satisfaction may erode work motivation, which in turn could compromise the efficiency and quality of health care provision.

Studies have explored the factors associated with physician perceived compensation satisfaction from 3 perspectives. The first considers physicians’ demographic characteristics, including educational background, years of work experience, and technical rank [4,5]. The second considers work hours, which are typically taken as representing physicians’ input [6]. The third considers physicians’ output, represented by financial compensation; this perspective most frequently focuses on the past [7,8]. These studies assume that the factors associated with physicians’ perceived compensation satisfaction are independent. However, these perceptions are complex and shaped by multifaceted comparative assessments captured in the Equity Theory. The Equity Theory was proposed by American psychologist J Stacy Adams in 1965, whose core purpose was to explain how individuals' perceptions of the fairness of reward distribution affect their work attitudes and behaviors [9]. The Equity Theory suggests that individuals think about their compensation by comparing their inputs with their outputs, as well as by comparing those inputs and outputs against others’ outputs [10,11]. Inputs include experience, skill, education, and effort, and outputs include basic salary, bonus, and benefits. When the input is perceived as exceeding the output, the perception of a need for greater compensation arises, and vice versa. Thus, the factors influencing this perception are not independent but interact to impact perception, and the compensation scheme is the basis for evaluating the relationship between input and output. To the best of our knowledge, few studies have investigated the combined effects of multiple influencing factors on physicians’ perceived compensation satisfaction. Furthermore, although it has been shown to deliver a competitive advantage in achieving employee satisfaction in the business field [12], little research has examined the influence of compensation scheme transparency in a physician context, considering physicians’ awareness of the compensation scheme, their level of understanding, and how promptly they are informed.

Our study attempts to address these gaps in the literature by examining the interaction of the factors influencing perceived compensation satisfaction along with compensation scheme transparency. Following previous studies, we include demographic characteristics, work hours, and compensation scheme transparency to represent alternative explanations for our research outcomes.

## Methods

### Study Design

We conducted a cross-sectional survey in China from April to May 2024 to collect the necessary data. To understand which factors or combinations thereof are most important for perceived compensation satisfaction, we used qualitative comparative analysis (QCA). Although QCA is traditionally associated with qualitative research, its application in this study aligns with a mixed methods approach.

### Procedure

We designed the questionnaire according to the aim of our study. First, we conducted a pilot survey on a small sample (n=10) in Beijing, Shandong, Hainan, Jiangsu, and Ningxia that included representatives of different cultures across North, South, East, and West China, respectively. The participants in the pilot surveys were colleagues or acquaintances of the researchers. We then revised the questionnaire according to the pilot test feedback. Pilot participants suggested clarifying terms such as compensation scheme fairness, leading to revised wording in the final questionnaire (eg, I think the department’s compensation scheme is fair). Second, our sample size meets the robustness requirements for QCA [13,14]; so, the research members tried their best to recruit more physicians to complete the questionnaire before data collection was scheduled to terminate. All questions were mandatory. The survey was anonymous, and the answers were sent directly to the database to ensure confidentiality.

### Ethical Considerations

This study was approved by the ethics committee of China-Japan Friendship Hospital (approval 2022-KY-218) and conducted in accordance with the World Medical Association Declaration of Helsinki. Informed consent was obtained from all participants via an online consent form on the questionnaire’s opening page (participants could only proceed after clicking “agree”), and all retained the right to opt out at any stage without adverse consequences. The survey was anonymous, with all participant data fully anonymized and deidentified. Participants who completed the survey received ¥1 in electronic compensation via WeChat, equivalent to US $0.14 at the time of data collection (April-May 2024).

### Measurements

The questionnaire consisted of three parts: sociodemographic characteristics (eg, sex, age, place of residence, education level, technical rank, specialty, and monthly salary), compensation scheme transparency, and perceived compensation satisfaction. The physicians rated compensation transparency and perceived compensation satisfaction on a 5-point Likert scale, with strongly disagree (score 1), disagree (score 2), neither agree nor disagree (score 3), agree (score 4), and strongly agree (score 5). Lower perceived compensation satisfaction was defined as an average score of less than 3 on the compensation perception questions. Higher perceived compensation satisfaction was defined as an average score equal to or greater than 3 on the compensation perception questions [15,16]. Cronbach α and the Kaiser-Mayer-Olkin value for the questionnaire were 0.955 and 0.924, respectively, indicating high total reliability and validity. All items loaded significantly on a single factor with loadings greater than 0.6, and the cumulative variance explained was 65.3%. These results collectively support the use of these constructs as unidimensional scales.

### Sociodemographic Characteristics

The questionnaire collected various sociodemographic characteristics of the physician participants to provide a comprehensive background for understanding their perceived compensation satisfaction. These characteristics included sex, categorized as male and female; age, recorded in years and further classified into several ranges (≤30, 31-40, 41-50, ≥51); education level, classified as bachelor and below, masters, and doctoral degree; technical rank, grouped into resident, attending physician, and (associate) professor; department, with specialties such as surgery, internal medicine, obstetrics and gynecology, pediatrics, traditional Chinese medicine, general practice, and emergency department; monthly salary, categorized into different ranges (≤¥3000; ¥3001-¥6000; ¥6001-¥10,000; ¥10,001-¥20,000; >¥20,000); and weekly work hours, classified as the average number of hours worked per week. All variables were collected via the online questionnaire.

### Compensation Scheme Transparency

Compensation scheme transparency was assessed through four questions: (1) in my opinion, I am very familiar with the compensation scheme; (2) when I want to learn about the compensation scheme, I can easily find out; (3) I am able to learn about the compensation scheme in a timely manner; and (4) senior leaders proactively share the compensation distribution details with me. The Cronbach α for this dimension was 0.947, indicating relatively high reliability.

### Perceived Compensation Satisfaction

On the basis of the comparative theory, perceived compensation satisfaction was assessed from multiple perspectives: external comparison, internal comparison, input–output comparison, expectation–reality gap, and compensation scheme perception. These were measured using 7 questions: (1) compared with other professions in the hospital, my compensation level is reasonable; (2) compared with physicians in other departments, my compensation level is reasonable; (3) compared with senior physicians in my department, my compensation level is reasonable; (4) compared with physicians of the same level in my department, my compensation level is reasonable; (5) my compensation is consistent with my efforts and contributions; (6) the actual compensation is in line with my expectation; and (7) I think the department’s compensation scheme is fair. The Cronbach α for this dimension was 0.944, indicating relatively high reliability.

### Sample Selection

We used the survey platform Questionnaire Star to host our questionnaire and distributed a generated link through WeChat. Each WeChat account could respond to the questionnaire only once (to avoid repeated responses) unless modified. Our trained research members were assigned to different geographical regions, including Beijing, Hainan, Shandong, Jiangsu, and Ningxia. Each sample hospital is a tertiary public hospital and has approximately 500 to 1000 physicians. The research members visited the sample hospitals to recruit physicians face-to-face through convenience sampling until the sample size was reached. Meanwhile, they explained the purpose of the study, informed the physicians of the anonymity of the survey, and stated that it would take about 10 minutes of their time if they were available. If the physicians agreed to participate in the survey, the research members would immediately present a QR code and help the physicians scanning it. The informed consent form was also on the opening page. Only after reading and clicking the “Agree” button could participants proceed to the questionnaire. Researchers were also available to respond to questions that prospective participants had regarding unclear terms or the questionnaire items. Compensation of ¥1 (US $0.14) was offered to participants who completed the survey. We also ensured a balance of sex, age, technical rank, and specialty to ensure the representativeness. The only participation exclusion was work experience of less than 6 months.

The response rate was approximately 80.2% (401/500). In total, 401 physicians responded to the survey. Among the questionnaires received, 76 were excluded owing to unreasonable response durations or logistic errors. Ultimately, 325 questionnaires were included in our analysis. Based on QCA recommendations, 10-80 cases are required for robust analysis. Our sample size of 325 far exceeds this threshold, ensuring sufficient statistical power.

### Statistical Analysis

SPSS software (version 24.0; IBM Corp) was employed to assess the reliability and validity of the questionnaire, conduct factor loading analysis, and compare the differences among groups.

As perceived compensation satisfaction has several complex influences rather than a single impact, we used fuzzy-set qualitative comparative analysis (fsQCA) to explore different combinations of factors. QCA was selected to analyze how combinations of factors jointly influence satisfaction. Traditional regression models assume linear relationships, whereas QCA identifies nonlinear interactions and multiple pathways to outcomes. This approach bridges qualitative and quantitative methods [17]. We used the fsQCA 3.0 software with a package developed by Charles Ragin and Sean Davey for this analysis.

We calibrated the raw data of the selected variables following previous research [14]. First, we determined the three calibration points that represented (1) fully belong, (2) the cross-point, and (3) fully do not belong. For this, we encoded the Likert scale data of strongly agree as fully belong, neither agree nor disagree as the cross-point, and strongly disagree as fully do not belong. Following the method of preset scale anchor points as thresholds [18,19], education level was set as 3, 2, and 1, and technical rank as 4, 2.5, and 1. Continuous variables were designated for 3 qualitative breakpoints (0.95, 0.5, and 0.05), the data were converted into values between 0 and 1 [20-22]. By setting these 3 thresholds, the original data were transformed into fuzzy scores ranging from 0 to 1 by using the Calibrate (x, n1, n2, n3) function in the fsQCA software.

The initial phase of QCA involves assessing the necessity of a singular condition as well as its absence for the full adoption of the phenomenon. A condition was considered sufficient when the consistency level exceeded 0.8. Furthermore, when the consistency level exceeded 0.9, this condition was deemed essential [20-24].

The next phase of QCA is configuration analysis, which was applied to our truth table to reveal the sufficiency of the perceptions associated with different configurations of multiple conditions. To calculate the complex, parsimonious, and intermediate solutions, the consistency threshold was set to 0.8 and the case frequency threshold to 1.

Statistical significance was assessed using *P* values, with a threshold of *P*<.05. Group differences (eg, technical rank, department, monthly salary) were analyzed via 2-sided chi-square tests for categorical variables and 2-sided *t* tests for continuous variables.

## Results

### Participant Characteristics

Of the 325 participants, 163 (50.2%) were males and 162 (49.8%) were females. The majority were aged either 31-40 years (131/325, 40.31%) or 41-50 years (133/325, 40.92%). The ratio of residents to attending physicians to (associate) professors was approximately 1:2:2.3. Among the physicians, 49.8% (162/325) earned more than ¥10,000 (US $1378) per month. The average weekly work hours were 52.30 (SD 12.26) hours. Table 1 presents the sociodemographic characteristics of the participants.

**Table 1:** Participant characteristics.

			Physicians with lower compensation satisfaction^a^ (n=133)		Physicians with higher compensation satisfaction^b^ (n=195)
**Sex, n (%)**					
	Male	59 (36.3)		104 (63.8)	
	Female	74 (45.7)		88 (54.3)	
**Age (y), n (%)**					
	≤30	19 (46.3)		22 (57.3)	
	31-40	56 (42.7)		75 (57.3)	
	41-50	52 (39.1)		81 (60.9)	
	≥51	6 (30)		14 (70)	
**Education level, n (%)**					
	Bachelor and below	46 (49.5)		47 (50.5)	
	Master	61 (38.6)		97 (61.4)	
	Doctorate	26 (35.1)		48 (64.9)	
	Technical rank				
	Resident	26 (41.9)		36 (58.1)	
	Attending	59 (48.8)		62 (51.2)	
	(Associate) Professor	48 (33.8)		94 (66.2)	
**Department, n (%)**					
	Surgery	38 (46.9)		43 (53.1)	
	Internal medicine	26 (34.7)		49 (65.3)	
	Obstetrics and gynecology	3 (30)		7 (70)	
	Pediatrics	9 (37.5)		15 (62.5)	
	Traditional Chinese medicine	9 (29)		22 (71)	
	General practitioner	16 (84.2)		3 (15.8)	
	Emergency department	10 (55.6)		8 (44.4)	
	Others	22 (32.8)		45 (67.2)	
**Monthly salary, n (%)**					
	≤¥3000 (US $≤412)	10 (71.4)		4 (28.6)	
	¥3001-¥6000 (US $413-$826)	19 (48.7)		20 (51.3)	
	¥6001-¥10,000 (US $827-$1378)	52 (47.3)		58 (52.7)	
	¥10,001-¥20,000 (US $1379-$2756)	42 (35)		78 (65)	
	>¥20,000 (>US $2756)	10 (23.8)		32 (76.2)	
Weekly work hours, mean (SD)			54.65 (13.35)		50.67 (11.19)
Perceived compensation scheme transparency, mean (SD)			2.43 (0.99)		3.71 (0.83)

^a^Lower compensation satisfaction was defined as an average score of less than 3 on the compensation perception questions.

^b^Higher compensation satisfaction was defined as an average score of equal to or greater than 3 on the compensation perception questions.

### Perceived Compensation Scheme Transparency

Overall, most physicians felt that their compensation schemes were transparent (see Table 2). The average score for perceived compensation transparency was 3.19 (SD 1.09). Their responses indicated that they had the means to learn more about the schemes and could do so promptly. They indicated that they were familiar with their compensation schemes and that their leaders were available to provide compensation details.

**Table 2:** Perceived compensation scheme transparency.

Item	Values, mean (SD)
In my opinion, I am very familiar with the compensation scheme.	3.18 (1.17)
When I want to learn about the compensation scheme, I can easily find out.	3.29 (1.18)
I am able to learn about the compensation scheme in a timely manner.	3.27 (1.16)
Senior leaders proactively share the compensation distribution details with me.	3.01 (1.20)

### Participants’ Perceived Compensation Satisfaction

As shown in Table 3, significant differences emerged in expectation–reality gap scores (2.70) compared with input–output comparisons (2.85; *P*<.05), suggesting that unmet compensation expectations drive dissatisfaction; the compensation gap within a department was perceived as being more reasonable than that among departments. The perceived compensation satisfaction was the lowest when compared with expectation, followed by the comparison of input and output.

**Table 3:** Physicians’ perceived compensation satisfaction.

Item			Values, mean (SD)
**External comparison**			
	Compared with other professions in the hospital, my compensation level is reasonable.	2.98 (1.04)	
	Compared with physicians in other departments, my compensation level is reasonable.	2.96 (1.04)	
**Internal comparison**			
	Compared with senior physicians in my department, my compensation level is reasonable.	3.18 (1.05)	
	Compared with the same-rank physicians in my department, my compensation level is reasonable.	3.47 (0.90)	
**Input-output comparison**			
	My compensation level is commensurate with my effort and contribution.	2.85 (1.13)	
**Expectation-reality gap**			
	The actual compensation I receive matches expected compensation.	2.70 (1.15)	
**Compensation scheme**			
	I think the compensation scheme is fair.	3.31 (1.04)	

### Factors Associated With Physicians’ Perceived Compensation Satisfaction

#### Necessity Analysis of Individual Conditions

Table 4 shows the test results of the necessary conditions for physicians’ perceived compensation satisfaction. Compensation scheme transparency was identified as a necessary condition for higher perceived compensation satisfaction, as indicated by its high consistency score of 0.901, which exceeds the conventional threshold of 0.9 [17].~Monthly salary and ~Weekly work hours were found to be sufficient conditions, with consistency scores of 0.805 and 0.803, respectively [25,26].

**Table 4:** Necessity test of the factors associated with physicians’ perceived compensation satisfaction.

Condition variable	Higher perceived compensation satisfaction			Lower perceived compensation satisfaction	
	Consistency	Coverage	Consistency		Coverage
Education level	0.704	0.620	0.579		0.711
~^a^Education level	0.672	0.533	0.691		0.765
Technical rank	0.681	0.642	0.574		0.756
~Technical rank	0.741	0.555	0.728		0.761
Working experience years	0.673	0.558	0.631		0.730
~ Working experience years	0.675	0.567	0.618		0.725
Weekly work hours	0.631	0.573	0.648		0.821
~Weekly work hours	0.803	0.621	0.663		0.715
Monthly salary	0.625	0.714	0.487		0.777
~Monthly salary	0.805	0.529	0.821		0.753
Perceived compensation transparency	0.901	0.799	0.525		0.650
~Perceived compensation transparency	0.606	0.477	0.838		0.922

^a^~ denotes the absence of a condition in fuzzy-set qualitative comparative analysis.

#### Adequacy Analysis of Conditional Configuration

As indicated in Table 5, three paths lead to higher perceived compensation satisfaction. The unique coverage for S1, S2, and S3 was 0.155, 0.019, and 0.109, respectively. Overall, these three paths show strong explanatory power owing to their good consistency (0.866) and relatively high coverage (0.709).

**Table 5:** Configuration analysis of the factors associated with perceived compensation satisfaction.

Conditions	Higher perceived compensation satisfaction^a^		
	Junior physicians (model 1)	Senior physicians (model 2)	
	S1 (one pathway)	S2 (pathway 1)	S3 (pathway 2)
Years of work experience	AC^b^	CC^c^	CC
Education level	CC	CC	IS^d^
Technical title	AC	IS	CC
Weekly work hours	IS	IS	AC
Monthly salary	IS	PC	AC
Compensation scheme transparency	PC^e^	PC	PC
Consistency	0.928	0.916	0.914
Raw coverage	0.434	0.399	0.435
Unique coverage	0.155	0.019	0.109

^a^Solution consistency=0.886; solution coverage=0.709.

^b^AC indicates absence of condition.

^c^CC indicates core condition.

^d^IS indicates insignificant.

^e^PC indicates the presence of a condition.

We merged the three paths into two to build models with greater explanatory power.

1. The first model is the junior physician model, which includes S1. Its basic expression is M1 = ~years of work experience × education level × ~technical title × compensation scheme transparency, that is, junior physicians care less about weekly work hours and salary when the compensation scheme is perceived as transparent. In this situation, they are satisfied with their compensation.
2. The second model is the senior physician model, which includes S2 and S3. The basic expression is M2 = years of work experience × compensation scheme transparency (education level × monthly salary × technical title × ~weekly work hours × ~monthly salary), that is, for senior physicians, a higher monthly salary and perceived compensation scheme transparency lead to higher perceived compensation satisfaction. If the salary is lower, a higher technical title and fewer weekly work hours combined with a transparent compensation scheme will drive perceived compensation satisfaction.

### Robustness Test

We set the consistency level from 0.8 to 0.85 and 0.73 to examine the robustness of the results. Compared with those before adjustment, the configuration paths, coverage, and consistency did not change substantially after adjustment. Thus, the results are robust.

## Discussion

### Principal Findings

QCA enables the analysis of complex causal configurations in quantitative data, making it suitable for exploring interactions between multiple factors influencing compensation satisfaction [17].

Through the application of QCA, we analyzed how combined factors influence physicians’ perceived compensation satisfaction. We found that compensation scheme transparency was a necessary condition for higher compensation satisfaction. Physicians who were more familiar with their compensation schemes, whether actively or passively, found it easier to learn about the scheme promptly and were more satisfied with their compensation. This finding aligns with a prior study by Chinese scholars investigating rural physicians' compensation satisfaction, which found that township hospital health care workers were not satisfied with their salary unless they were aware of the salary scheme design [27]. Research conducted in the United States has found that the gender salary gap among surgeons led to burnout among female surgeons, and while compensation transparency reforms narrowed this gap, disparities persisted. Consequently, the researcher promoted a transparent, performance-based, and objective compensation plan [28,29]. In China, physician compensation comprises fixed and variable components. The fixed part, which is primarily determined by years of work experience and one’s professional title, constitutes a minor proportion of the total compensation with narrow disparities among physicians. The variable part, typically based on performance, including clinical workload, medical quality, patient satisfaction, and academic research outputs, ranges from 50% to 70% of total compensation [30,31]. Therefore, regarding compensation scheme transparency, the most important aspect is the variable part design and associated feedback. To improve physicians’ perceived compensation satisfaction and work behavior, hospital managers should maintain transparent performance assessment criteria and provide clear performance feedback.

The determinants of compensation perception were heterogeneous between junior and senior physicians. For junior physicians, if the compensation scheme is transparent, they would be satisfied even with lower pay or longer working hours. In contrast, they prioritized the transparency of the compensation scheme. For senior physicians, transparent compensation combined with a relatively high salary contributed to high compensation satisfaction. In the case of a lower salary, senior physicians’ satisfaction was promoted by a transparent compensation scheme, a higher technical title, and fewer work hours. For junior physicians, neither salary nor work hours seemed to be significantly associated with their compensation satisfaction. Therefore, junior physicians differ from other young adults at a similar career stage, who are particularly interested in higher salaries and work-life balance and unwilling to pay more than a minor amount for training to enhance their skills and knowledge [32,33]. The difference may originate from the fact that the salary of physicians increases over time with training [34]. Furthermore, the unique nature of the medical profession and the complexity of medical knowledge could explain why junior physicians do not feel the need to pursue higher salaries at this stage of their career. Moreover, junior physicians are eager to acquire more professional skills to enhance their competence in the field [35], and they understand that this initially requires long work hours and a lower salary. A survey of junior physicians in Sudan has found that their training program served as a nonmonetary incentive to trade off portions of their salaries [36]. Given the priority on professional skills, training plans offered to junior physicians should be carefully designed, and measures to address supervision-related issues are recommended in the short term from a cost perspective. For senior physicians, in addition to compensation scheme transparency, monthly salary, technical rank, and work hours all combined to affect compensation satisfaction. High entry-level requirements for physicians regarding education, training, and certification are common worldwide. Thus, we may hypothesize that high entry barriers would lead to higher salaries [37-39]. Logically, physicians should earn a relatively high compensation, as their salary is an essential component of their occupation that enables them to fulfil family responsibilities. Our findings are in line with previous research reporting that higher salaries can lead to higher satisfaction and stimulate employee enthusiasm for work [40,41]. Chinese scholars have found that low salaries relative to high training costs and low compensation for medical staff strongly incentivize overtreatment, contributing to increased medical service expenditures [42]. Given the entry-level requirements and low compensation among Chinese physicians, increasing their salaries is a significant incentive to ensure their satisfaction and avoid opportunistic behavior.

Additionally, we found that physicians with a higher technical rank and fewer work hours had higher compensation satisfaction, even when their salaries were low. This higher satisfaction with compensation may stem from Maslow’s Needs Theory, which suggests that the highest level of need is the realization of self-worth, and once the needs of a higher level are fulfilled, the more basic needs such as salary become less important [43,44]. Regarding technical rank, a survey conducted in 161 US universities also reported that associate professors were less satisfied in their jobs than full professors [45]. Thus, the title of professor serves as an approval of worth and could be the goal for many physicians. Therefore, to ensure higher compensation satisfaction, hospital managers should pay attention to the technical promotion pathway for physicians. Notably, due to the cross-sectional design, the next step will require a cohort study to validate the findings of this research.

### Limitations

This study has limitations. As the physicians were selected through convenience sampling, the nonrandomness of this technique could have introduced limiting factors. Although we strictly controlled sex, age, technical rank, and specialty distribution to ensure sample balance across these four key aspects, the inherent nature of convenience sampling still limits sample representativeness, and the generalizability of the conclusions requires further validation. For example, the samples were mainly from physicians willing to participate in the survey at sampled hospitals, which may lead to self-selection bias. Meanwhile, the results may be overestimated or underestimated because of the self-reporting design of our questionnaire. There are some potential influencing factors such as occupational risk that should be considered. Future research is required that employs more rigorous sampling methods, incorporates larger and more diverse samples across multiple institutions, and accounts for more potential influencing factors—all of which would enhance the generalizability of our findings and the stability of our results.

### Conclusions

Our study considered the dynamic effects of multiple factors on physician compensation satisfaction and provided management advice to hospitals and other institutions looking to retain personnel. We found that the factors associated with compensation satisfaction differed between junior and senior physicians. For junior physicians, compensation scheme transparency, not salary level, influenced their perceived compensation satisfaction. For senior physicians, salary level was an important factor but not consistently the only decisive factor influencing satisfaction. A higher technical rank could offset the adverse effects of lower salaries. Therefore, hospital managers should focus on improving the transparency of compensation schemes and customizing benefit packages for specific employees.

## Data Availability

Study data are available from the corresponding author upon reasonable request.

## Abbreviations

fsQCA: fuzzy-set qualitative comparative analysis
QCA: qualitative comparative analysis
